# Supplementary material for: Preventing the Increase in Lysophosphatidic Acids: A New Therapeutic Target in Pulmonary Hypertension?
Source: Metabolites. 2021 Nov 17;11(11):784. doi: 10.3390/metabo11110784 (PMC8621392; doi:10.3390/metabo11110784)
Supplement: Supplementary file 1 [file metabolites-11-00784-s001.zip › metabolites-1453337-supplementary/Supplementary File S1 - Analytical method validation.pdf]

Supplemental Figure S1 :

Chromatograms of rat plasma showing the elution of LPA (A), LPC (B) LPE (C) and MAG (D).

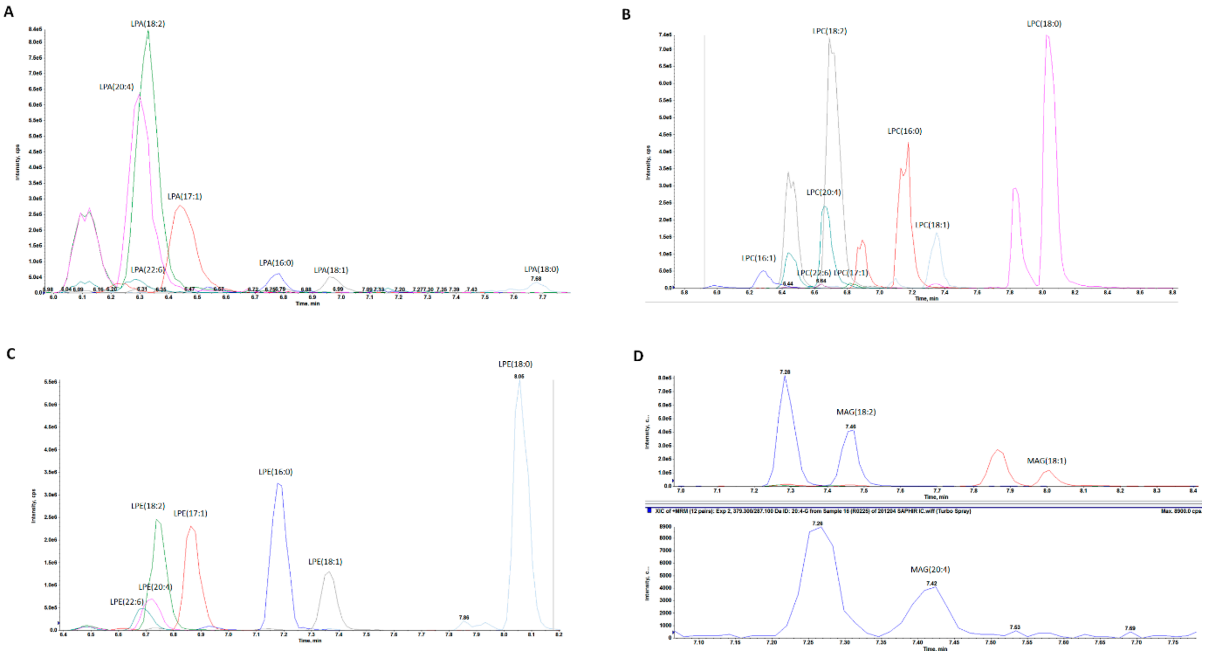

LPA: lysophosphatidic acids; LPC: lysophosphatidylcholine; LPE: lysophosphatidylethanolamine; MAG: monoacylglycerol. All compounds exhibited two peaks corresponding to the position of the esterified fatty acid (1- or 2-) on the glycerol backbone. Annotation corresponds to the integrated compounds (1-acyl lysophospholipid released after the action of phospholipase A_2).

Supplemental Figure S2 : Calibration curves of the analytical standard of LPA(16:0) (r^2=0.998, A), LPA(18:1) (r^2=0.999, B), LPC(16:0) (r^2=0.995, C), LPC(18:1) (r^2=0.995, D), LPE(16 :0) (r^2=0.990, E) and MAG(18:1) (r^2=0.999, F) performed in a surrogate matrix (sodium chloride 0.9%)

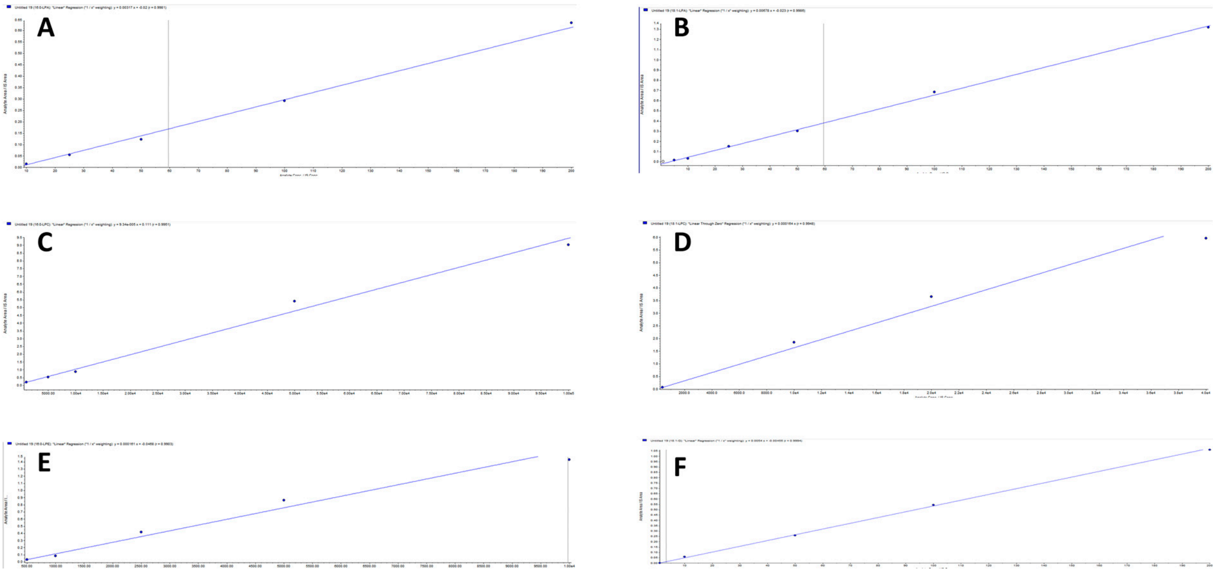

LPA: lysophosphatidic acids; LPC: lysophosphatidylcholine; LPE: lysophosphatidylethanolamine; MAG: monoacylglycerol.

Supplemental Table S1: Repeatability and reproducibility analysis using pooled plasma. Analyses were performed in triplicate on two separate days using compound-to-IS normalized area under curve.

**Analyte.**	**Day 1**<br>**Mean ± sd (CV%)**	**Day 2**<br>**Mean ± sd (CV%)**	**Overall**<br>**Mean ± sd (CV%)**
LPA(16:0)	0.221 ± 0.010 (4.5)	0.197 ± 0.028 (14.1)	0.209 ± 0.023 (11.0)
LPA(18:0)	0.177 ± 0.020 (11.5)	0.154 ± 0.023 (14.8)	0.166 ± 0.024 (14.1)
LPA(18:1)	0.121 ± 0.007 (5.6)	0.120 ± 0.017 (14.0)	0.120 ± 0.011 (9.54)
LPA(18:2)	2.05 ± 0.07 (3.3)	2.11 ± 0.26 (12.3)	2.08 ± 0.17 (8.34)
LPA(20:4)	1.69 ± 0.077 (4.5)	1.94 ± 0.42 (21.9)	1.81 ± 0.30 (16.8)
LPA(22:6)	0.150 ± 0.007 (4.8)	0.174 ± 0.032 (18.5)	0.162 ± 0.025 (15.2)
LPC(16:0)	16.2 ± 2.1 (13.3)	19.2 ± 2.0 (10.4)	17.7 ± 2.5 (14.2)
LPC(16:1)	2.69 ± 0.37 (13.7)	3.43 ± 0.43 (12.4)	3.06 ± 0.54 (17.6)
LPC(18:0)	23.2 ± 3.4 (14.6)	23.5 ± 3.6 (19.5)	23.4 ± 3.6 (15.5)
LPC(18:1)	6.28 ± 0.94 (15.0)	6.58 ± 1.14 (17.3)	6.43 ± 0.95 (14.7)
LPC(18:2)	28.1 ± 1.99 (7.1)	27.0 ± 5.0 (18.5)	27.5 ± 3.44 (12.5)
LPC(20:4)	9.25 ± 1.96 (21.2)	9.27 ± 1.84 (19.8)	9.26 ± 1.70 (18.3)
LPC(22:6)	0.410 ± 0.088 (21.3)	0.512 ± 0.211 (48.9)	0.461 ± 0.155 (33.7)
LPE(16:0)	0.828 ± 0.019 (2.3)	0.776 ± 0.114 (14.7)	0.802 ± 0.079 (9.8)
LPE(18:0)	1.27 ± 0.49 (38.5)	1.38 ± 0.33 (23.8)	1.33 ± 0.38 (28.5)
LPE(18:1)	0.404 ± 0.030 (7.5)	0.404 ± 0.038 (9.4)	0.404 ± 0.031 (7.6)
LPE(18:2)	0.424 ± 0.095 (22.3)	0.369 ± 0.036 (9.8)	0.397 ± 0.071 (17.9)
LPE(20:4)	0.208 ± 0.031 (15.0)	0.134 ± 0.015 (11.3)	0.171 ± 0.04 (26.9)
LPE(22:6)	0.123 ± 0.024 (19.8)	0.102 ± 0.031 (29.8)	0.113 ± 0.028 (24.2)
MAG(18:1)	0.094 ± 0.029 (30.2)	0.079 ± 0.009 (11.2)	0.087 ± 0.021 (23.8)
MAG(18:2)	0.671 ± 0.151 (22.5)	0.550 ± 0.085 (15.5)	0.610 ± 0.128 (21.0)
MAG(20:4)	0.0085 ± 0.0016 (18.9)	0.0070 ± 0.0023 (32.9)	0.0078 ± 0.0020 (25.2)

CV : coefficient of variation (expressed in %); IS: internal standard; LPA: lysophosphatidic acids; LPC: lysophosphatidylcholine; LPE: lysophosphatidylethanolamine; MAG: monoacylglycerol ; sd: standard deviation.
